# Unlocking Genetic Mysteries during the Epic Sperm Journey toward Fertilization: Further Expanding *Cre* Mouse Lines

**DOI:** 10.3390/biom14050529

**Published:** 2024-04-28

**Authors:** Pengyuan Dai, Chaoye Ma, Chen Chen, Min Liang, Shijue Dong, Hao Chen, Xiaoning Zhang

**Affiliations:** Institute of Reproductive Medicine, Medical School, Nantong University, Nantong 226001, China; pengyuandai@ntu.edu.cn (P.D.); 2113310057@stmail.ntu.edu.cn (C.M.); 2113510009@stmail.ntu.edu.cn (C.C.); 2013510008@stmail.ntu.edu.cn (M.L.); 2113310047@stmail.ntu.edu.cn (S.D.); chenhao@ntu.edu.cn (H.C.)

**Keywords:** *Cre* recombinase, *Cre*/*LoxP*, conditional knockout, germ cells, testes, epididymis

## Abstract

The spatiotemporal expression patterns of genes are crucial for maintaining normal physiological functions in animals. Conditional gene knockout using the cyclization recombination enzyme (*Cre*)/locus of crossover of P1 (*Cre*/*LoxP*) strategy has been extensively employed for functional assays at specific tissue or developmental stages. This approach aids in uncovering the associations between phenotypes and gene regulation while minimizing interference among distinct tissues. Various *Cre*-engineered mouse models have been utilized in the male reproductive system, including *Dppa3*-*MERCre* for primordial germ cells, *Ddx4*-*Cre* and *Stra8*-*Cre* for spermatogonia, *Prm1*-*Cre* and *Acrv1*-*iCre* for haploid spermatids, *Cyp17a1*-*iCre* for the Leydig cell, *Sox9*-*Cre* for the Sertoli cell, and *Lcn5/8/9*-*Cre* for differentiated segments of the epididymis. Notably, the specificity and functioning stage of *Cre* recombinases vary, and the efficiency of recombination driven by *Cre* depends on endogenous promoters with different sequences as well as the constructed *Cre* vectors, even when controlled by an identical promoter. *Cre* mouse models generated via traditional recombination or CRISPR/Cas9 also exhibit distinct knockout properties. This review focuses on *Cre*-engineered mouse models applied to the male reproductive system, including *Cre*-targeting strategies, mouse model screening, and practical challenges encountered, particularly with novel mouse strains over the past decade. It aims to provide valuable references for studies conducted on the male reproductive system.

## 1. Introduction

**Figure 1:**
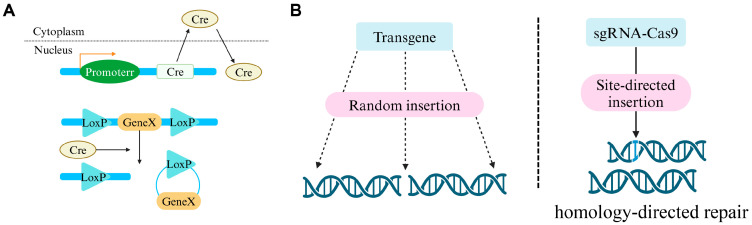
(**A**) DNA excising mediated by the *Cre*-*LoxP* system. The mouse DNA was flanked with *LoxP* sites. *Cre* catalytic activity was driven by the exogenous promoters and removed the target exon between *LoxP* sites after *Cre* recombinase translocated into the nucleus. (**B**) Two kinds of gene recombination modes. (1) Gene integration via a conventional transgenic approach was inserted into the host genome randomly with the unpredictable recombination site and multiple integrations (left panel); (2) Cas9 was directed to the target locus and cut double-stranded DNA after binding to sgRNA. The exogenous genes were knocked-in by homology-directed repair efficiently and inherited stably (right panel).

Conditional knockout (cKO) with recombinant enzymes allows for the removal of targeted genes to investigate their functions in physiological processes. Recombinase systems, including the cyclization recombination enzyme/locus of crossover of P1 (*Cre/LoxP*), v*Cre*/v*LoxP*, s*Cre*/s*LoxP*, *Flp*/*FRT*, *Dre*/*Rox*, and *Vika*/*Vox* [1,2,3,4,5], are utilized with *Cre*/*LoxP* being the most prevalent. *Cre*, a 38-kDa recombinase with site specificity, has been an in vivo molecular tool for over 30 years [6,7]. It recognizes DNA sequences between two *LoxP* sites (Figure 1A). The target DNA sequences flanked by *LoxP*, engineered artificially, are termed “floxed” and can be specifically ablated by *Cre* driven by the promoters of cell-specific genes after recombining downstream of the promoters via transgenic technology or knock-in (KI) into endogenous genic loci [8,9]. However, in most cases, the promoter in *Cre*-constructed vectors is incomplete and lacks distal regulatory elements (e.g., enhancers), which further reduces transcription efficiency (Figure 1B). In addition, the randomness of genomic insertions can result in uncontrollable recombination sites [10] and may exert toxic effects when multiple copies are expressed in cells [9]. Notably, as the homozygous ectopic expression of *Cre* lines behaves more unpredictably, the heterozygous *Cre*-engineered mouse was generally crossed with the flox-transgenic mouse, significantly decreasing breeding efficiency. Furthermore, the KI approach facilitates gene insertion into well-defined genic loci [11]. The CRISPR/Cas9 system, a DNA targeting and editing approach, has been broadly employed to establish *Cre* recombination mouse models driven in situ by target promoters after site-directed *Cre* insertion [12]. This method requires less time and is less costly for generating the desired transgenic mouse lines [13]. Functional gene investigation employing *Cre* transgenic mouse strains generated by CRISPR/Cas9 is more reliable and repeatable because the insertion locus is clear, promising endogenous gene expression away from interference to a large extent [13] (Figure 1B).

**Figure 2:**
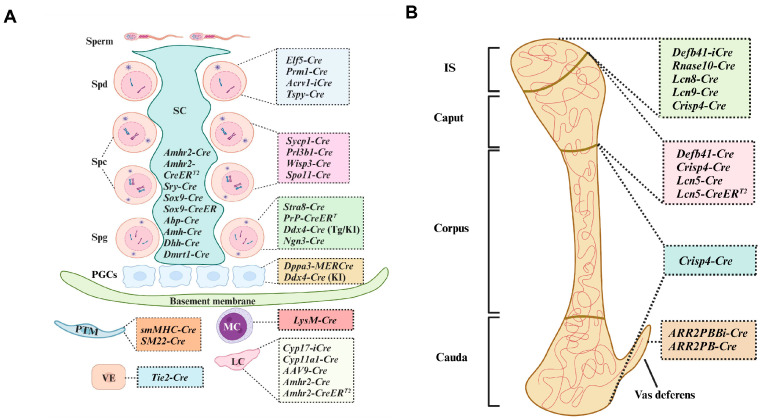
Schematic diagram showing the established novel mouse strains and *Cre* lines used widely in the male reproductive system. (**A**) Cre models in the testes specific for each primary cell type, including PTM and peritubular myoid cells. SC, Sertoli cell; LC, Leydig cell; PGCs, primordial germ cells; Spg, spermatogonia; Spc, spermatocyte; Spd, spermatid; VE, vascular endothelial cells; MC, myeloid lineage cell (**B**) The *Cre* lines specific for IS, caput, corpus, cauda, and vas deferens.

Spermatogenesis, which occurs in seminiferous tubules as the foundation of male fertility, is a complex process that produces mature gametes. It involves spermatogonia proliferation and differentiation into spermatocytes, spermatid generation through meiosis, and spermatozoa release into the tubule lumen after spermiogenesis [14]. Any aberration in these processes may impede fertilization. A systematic and in-depth investigation of the mechanisms regulating the orchestrated stages of spermatogenesis is crucial for treating male infertility or, conversely, the design of male contraceptives that target the desired developmental stages. *Cre*/*LoxP* recombination has been extensively utilized in specific DNA modifications targeting spermatogenic cells, such as *Ddx*4-*Cre* [15] for spermatogonia, *Sycp1*-*Cre* [16] for spermatocytes, and *Prm1*-*Cre* [17] for spermatids. Hammond et al. (2009) [18] and Smith et al. (2011) [19] reviewed genetic tools, including *Cre* recombinases specific for male and female germ cells and markers used to characterize cellular behavior or purify living germ cells. However, many subsequent reports with conditional ablations using these *Cre* have shown that they are not as specific or efficient as previously thought. Issues such as the efficiency and specificity of *Cre* recombinase not aligning with presuppositions need to be addressed given the widespread availability of abundant *Cre*-engineered lines, including heterotopic expression of non-target tissues [20,21], leakage [22], the instability of KO efficiency [17,23], and global KO [15,24]. Furthermore, various novel *Cre* mouse lines have been engineered with the request for more detailed dissection in critical cellular processes during spermatogenesis over the past decade (Figure 2). In this review, we summarize the novel advances in these events, especially the removal of targets in the primordial germ cells (PGCs), testis, epididymis, prostate, and seminiferous duct, as well as the unintended consequences of crossing with *Cre* mouse lines to provide a referential basis for studies investigating the functional roles of genes in male reproduction.

## 2. *Cre* Lines for PGCs and Spermatogonia

### 2.1. Ddx4-Cre

*Ddx4*, also known as *Vasa* or *Mvh*, is located on mouse chromosome 13 and is an ATP-dependent RNA helicase that is highly conserved in mammals, belonging to the DEAD (Asp-Glu-Ala-Asp) box family [25]. *Ddx4* is primarily detected in germ cells with lower levels in other tissues such as the thymus and pancreas [26], appearing as early as embryonic day 10.5–12.5 (E10.5–12.5) and persisting in both the testes and ovaries throughout life [27,28]. Male infertility ensues after *Ddx4* KO [27], while the *Ddx4* mutation has minimal influence on female fertility, although oocytes with elevated levels of *Ddx4* have been observed [29]. *Ddx4*-*Cre* lines (Strain #:006954) are available for functional analysis, particularly concerning genes implicated in spermatogonial establishment, maintenance, and differentiation. Notably, *Ddx4*-*Cre*-mediated target recombination efficiently initiates at E15, as indicated by *the Rosa26*-*lacZ* reporter, but it was inconsistent with its endogenous expression [30]. Recently, an improved *Cre* (*iCre*) line targeted by CRISPR/Cas9 exhibited higher efficiency in PGCs, with *Cre* activity initiated as early as E10.5. Furthermore, the probability of descendants with global KO from paternal *Cre* carriers was lower than that of *Ddx4*-*Cre* lines [30] for special floxed alleles, such as *Cripto^flox^* [15]. However, both types of *Cre* carriers in females can lead to offspring with global KO, demonstrating a robust maternal effect [15,30]. Moreover, reports on the minimal ectopic activity of *Ddx4*-*Cre*/*iCre* recombinase have surfaced [15,30].

*CreER* constructs have been used for temporal gene ablation, with *Cre* fused to the mutated human estrogen receptor (ER) and named *CreER* [31]. *CreER^T^*, as a human *ER* variant, was more sensitive to 4-hydroxytamoxifen (4-OHT), the metabolite of tamoxifen (TAM), compared to endogenous 17β-estradiol [32,33]. The mutants of *CreER^T1^* and *CreER^T2^* display even higher sensitivity to 4-OHT [34]. Moreover, *CreER^T2^* has been used extensively due to its robust activation at a specific stage upon TAM treatment with minimal background in *Cre* activity [34]. Given the unknown insertion site of *Ddx4*-*CreER^T2^* acquired through random gene recombination, Hoai et al. established a novel bicistronic mouse strain via CRISPR/Cas9, known as B6-*Ddx4*^em1(*CreERT2*)Utr^ [20]. However, infertility was found in the homozygous mouse line, manifesting as spermiogenesis arrest and absent mature spermatozoa. The ATPase activity of *Ddx4* was halved in homozygotes, possibly due to the additional 25 amino acid residues introduced by the *P2A* sequence, which protects *Ddx4* from being knocked out. Additionally, *Cre* recombination is primarily activated in the testes and ovaries but not in the pancreas or thymus [20]. In summary, novel-inducible B6-*Ddx4^em1(CreERT2)Utr^* mice are available for studying sterility at specific spermatogenic stages, and the use of the heterozygote *Cre* line during mating is recommended.

### 2.2. Stra8-Cre

Stimulated by retinoic acid 8 (*Stra8*), localized on mouse chromosome 6, it is specifically expressed in germ cells and is essential for initiating meiosis during spermatogenesis and oogenesis, the deficiency of which leads to abnormal chromosomal behavior [35]. In females, *Stra8* expression coincides with oocyte meiosis initiation at E13, with the peak at E14.5, and declines sharply after E16.5 [36]. In males, *Stra8* expression is detected at 5 days post-parturition (DPP), with reports also indicating expression at 3 DPP, persisting in preleptotene spermatocytes [37]. As early as 2008, the *Stra8*-*Cre* line (Strain #:008208) was generated to study male germ cell development with a recombination efficiency of >95%, whereas the *Cre* line did not work in transgenic female mice [38]. The diverse strategies of mating with *Stra8*-*Cre* resulted in different efficiencies and offspring phenotypes. Bao et al. constructed an *iCre* regulated by the *Stra8* promoter, excising the *Mov10l1* flox allele with high efficiency in spermatogenetic cells. However, it failed to remove two *Mov10l1* flox alleles in single breeding, and the testicular phenotypes were less severe compared to completely knocked-out mice [39]. Additionally, Balnco et al. observed the presence of the DOLT1 protein in offspring born to mice with a single allele of *Dotl1* excised by the *Stra8*-*Cre* recombinase line [40]. This discrepancy could stem from the transgenic mice driving *Cre* expression using only part of the *Stra8* promoter, which differs from the endogenous *Stra8* expression profile. The uncertain insertion site and varying copies of *Cre* may also result in lower KO efficiency. Recently, the functional responsive elements in the 2.9 kb promoter lying upstream of 1.4 kb promoters that promised optimal *Stra8* expression in vivo were characterized [41]. Therefore, *Cre* inserted into the targeted locus of *Stra8* may exhibit optimal recombinase activity. The CRISPR/Cas9-mediated targeted mutation was employed to insert *Cre* or *EGFP*-*Cre* at the stop codon of *Stra8* to study specific genes involved in spermatogenesis [42]. Avery et al. knocked in engineered *P2A*-*Cre* via the CRISPR/Cas9 system and recombinase activity without disturbing *Stra8* expression or mouse fertility. It is worth noting that the *Stra8*-*P2ACre* line also exhibited *Cre* activity in female mouse germ cells at E12.5–E16.5 [43]. Xue et al. generated germline-specific *Nat10* cKO females using *Stra8*-*GFPCre* or *Zp3-Cre* KI and revealed *Nat10*-indispensable oocyte meiosis progression, growth, and maturation [44]. The *Stra8*-*Cre* female strain constructed by targeted insertion contributes to studies focusing on oogenesis; however, offspring suffering from global KO appeared upon mating with the female *Stra8*-*Cre* line because of the long half-life of *Stra8*-*Cre* expressed in the oocyte.

### 2.3. PrP-CreER^T^

*Prp* is primarily localized in the central nervous system (CNS) and shows low expression in the spleen, kidney, heart, and testes [45]. Specific *PrP* fragments or reporter genes were engineered for expression in the CNS. Philipp et al. reported that one of the transgenic lines expressing *CreER^T^* induced by TAM and driven by 7.5 kb *PrP* fragments was restricted to the testis, with recombination occurring in particular in the spermatogonia, spermatocytes, and spermatids [46]. Following treatment with TAM for 6 days, approximately 30% of the floxed alleles underwent recombination in the testis without ectopic expression in other tissues, and even after 6 weeks of induction, 50% of seminiferous tubules still showed *Cre* activity [46]. Despite lower recombinant activity, this mouse strain provides a relatively specific tool for the genetic analysis of germ cells.

### 2.4. Dppa3-MERCre

*Dppa3* (also named *Stella* or *Pgc7*) marks primordial germ cells emerging at E7.25, with robust expression persisting at around E15.5 in males and E13.5 in females [47,48]. *Dppa3* is also expressed from the zygotic stage onward and remains in all blastocyst cells at E4.5 [48,49]. A novel transgenic mouse line (*Dppa3*-*MERCre*) was generated by Takayuki et al., and *Cre* recombinase expression was regulated by the *Dppa3* promoter under simultaneous induction with 4-OHT, binding to domains flanking the *Cre* sequence [50]. Additionally, these strains demonstrated specific and efficient KO outcomes in PGC development, preimplantation embryos, and oocyte growth after 4-OHT stimulation [50]. Thus, *Dppa3*-*MERCre* can serve as a strain for exploring gene function during germ cell lineage development. Inducible KO also contributes to defining the function of specific genes causing embryonic lethality after target silencing. However, determining the optimal dose of the inductive agent before treatment in pregnant models is crucial to avoid miscarriage or other toxic effects associated with the drug.

### 2.5. Eomes-CreER^T^

Eomesodermin (EOMES) is a T-box transcription factor crucial for intrinsic functions in immune cell development [51]. In addition, *Eomes* is vital for gastrulation formation and trophoblast development. Mutation in *Eomes* blocks at the blastocyst phase, preventing differentiation into trophoblasts after *Eomes* inactivation in the trophectoderm, suggesting its potential role in trophoblast stem cell physiology [52]. Stefanie et al. established an inducible *CreER^T^* that carried 4-OHT in the MEFs sequence at the *Eomes* locus (*Eomes*-*CreER^T^*) to study *Emoes* expression in pluripotency reprogramming in adult somatic mammalian cells [53]. Moreover, *Eomes* is localized in a subset of undifferentiated spermatogonia and contributes to regeneration after chemical injury and spermatogenesis regulation [54]. This suggests its potential to uncover target functions by establishing *CreER^T^* recombination in germ cell lines via in situ TAM induction.

### 2.6. Ngn3-Cre

NGN3 is a marker of postnatal undifferentiated spermatogonial cells from 3 DPP [55] and is localized in the developing CNS, adult enteroendocrine cells, and pancreas [56]. Two *Ngn3*-*Cre* mouse strains were established for functional research on SSCs and spermatogenesis. Originally, *Cre* recombinase was constructed on an artificial bacterial chromosome (BAC) and inserted into the mouse *Ngn3* locus 23 kb region [57]. A strong recombination signal was observed in the seminiferous tubules at 7 DPP, corresponding to the start of endogenous *Ngn3* expression [57], with an efficiency close to 100% observed in juvenile and adult testes [57,58,59]. Another *Ngn3-Cre* mouse line (Strain #:005667) was generated by inserting a *Cre* recombinase cassette next to the start codon of the endogenous *Ngn3* [60], with *Cre* catalytic activity appearing at 7 DPP [61,62] (Figure 2). Studies focusing on the action of SSCs [63,64] and germline development [65] during spermatogenesis have been conducted using this transgenic strain. Given the multiple localizations of NGN3 in the pancreas, CNS, and enteroendocrine cells, spatial specificity is gaining increasing attention.

### 2.7. Other Cre Lines for PGCs and Spermatogonia

Other *Cre* lines available for functional studies of PGCs and spermatogonial targets are described in Table 1. *Tnap*-*Cre* in PGCs was specifically designed to excise targets at E9.5–E10.5, while broad expression patterns were detected in the neural tube, placenta, labyrinthine region, and intestine after mid-gestation, achieving an efficiency of approximately 50% [66]. *Nanos3* localizes to migrating PGCs and stages after settling in the gonads of both males and females. *Nanos3*-*Cre* bioactivity was observed at E7.75, with an efficiency ranging from 11 to 25% [67]. *Blimp1*-positive cells considered the progenitors of PGCs in both sexes, were expressed persistently from E6.25 to E13.5 [68,69]. *Blimp1*-*Cre* was found to be activated at E7.8 in PGCs, with a positive ratio of 55−78% [68]. However, as *Blimp1* is also expressed in the retina, limbs, heart, pharynx, and B and T lymphocytes [70], *Blimp1*-*Cre* may not be suitable for all functional experiments. Tamoxifen-inducible ubiquitin C (*UBC*)-*CreER^T2^* [71,72] and *ROSA26*-*CreER^T2^* [73] were utilized for special target ablation in adult spermatogonia post-tamoxifen administration, showing high efficiency without detectable *Cre* recombination activity in somatic cells of the testis and other germ cells. Nonetheless, *UBC*-*CreER^T2^* activity was found in the somatic cells of 4-week mice testes, indicating an age-dependent *Cre* recombinase property [74,75]. Considering the broad expression of *UBC* and *ROSA26*, the predicted SSC phenotypes may be affected by functional defects in other tissues. *Cre* fused with the mouse mutant *ER* ligand-binding region is known as *MerCreMer* [76]. *Oct-4* in PGCs starts at E7.5 in developmental embryos [77]. The *Oct4*-*MerCreMer* mouse line was used to perform the genetic analyses of undifferentiated spermatogonia, including As, Apr, and Aal types [78,79]. However, because *Oct4* is expressed in various tissues during postnatal development, such as the skin, liver, and pancreas [80], the cKO phenotype should be evaluated to exclude other disturbances.

## 3. *Cre* Recombination Lines for Spermatocytes

### 3.1. Sycp1-Cre

Synaptonemal complex protein 1(SYCP1), primarily constituting the synaptonemal complex in meiosis, mainly functioned in recombination and XY body formation during leptotene to early pachytene stages [81]. Vidal et al. generated *Sycp1*-*Cre* mouse lines (Strain #:003466) and verified the recombination efficiency in mice harboring *Sycp1*-*Cre* and another transgene flanked by *LoxP*. They found that descendants of male double-transgenic mice mating with normal females were widely excised from the *LoxP*-flanked sequence. Even males were hemizygous for *Sycp1*-*Cre*, with the targets being removed in offspring without the *Cre* gene, indicating that recombination occurred during paternal spermatogenesis [16]. Additionally, the *Sycp1*-*Cre* driving target KO was restricted in the testis exclusively, and the flox-flanked segments were never altered in female progeny [16], likely due to the heterotopic transgenic expression initiated by partial promoters of *Sycp1*. Sanny et al. also confirmed that the specificity of *Sycp1*-*Cre* was restricted in the testes by crossing with *R26R* reporter transgenic mice, with recombination occurring in zygotene spermatocytes. Higher KO efficiency was observed in the entire meiosis stage, indicating that the *Sycp1*-*Cre* line is suitable for gene analysis during the germinal differentiation process [82].

*Sycp1*-*Cre* recombination activity decreased in the second generation during meiosis. This defect was associated with cytosine methylation, occurring in *LoxP* and transgenic sequences and extending to longer sequences in chromosomes. The allelic locus was also affected by a structure similar to the transvection defined in *Drosophila* [22]. In addition, Reza et al. created germ cell lines that were conditional knocked-out *Ikkβ* through mating with *Sycp1*-*Cre* mice; however, approximately 43% of the *LoxP*-flanked sequence was recombinant in offspring due to the epigenetic modification of *LoxP* in fourth- and fifth-generation (F4 and F5) mice [83]. The sodium bisulfite treatment sequencing revealed that methylated *LoxP* site cytosines were only found in F4 or F5 mice rather than in re-derived F1 *Ikkβ^fl^*^/^*^fl^* mice unmated with *Sycp1*-*Cre*. The methylated *LoxP* sites were derived from *Sycp1*-*Cre*-sired IKKβ-*LoxP* alleles in paternal lines, while maternal *LoxP* was unmethylated [83]. Despite its suitability for gene analysis during germinal differentiation, *Sycp1* presents epigenetic modifications and low recombination efficiency that cannot be neglected. 

### 3.2. Prl3b1-Cre

Prolactin family 3, subfamily B, member 1 (*Prl3b1*) is localized in mouse chromosome 13, and its expression has been detected in the nervous system, eye, and epithelium of the digestive tract in mid-to-late developing embryos [84]. *Prl3b1* is found in testicular germline cells, particularly in spermatocytes and haploid spermatids [85]. The *Cre* recombinase mouse line under the control of a 2.5 kb *Prl3b1* promoter (*Prl3b1-Cre*) was generated, and efficiency and specificity analyses were conducted by mating with *R26GRR* mice expressing the fluorescent protein, tDsRed, after *Cre* activation [85]. *Cre* recombination activity was determined to be limited to the testis, epididymis, and seminiferous ducts. A strong fluorescence signal was observed from elongated spermatids and spermatozoa in seminiferous tubules, with 74% recombination efficiency detected in germ cells after in vitro fertilization [85]. In summary, the *Prl3b1*-*Cre* strain offers a powerful tool for investigating the genes involved in the spermatogenic process, owing to its robust specificity in germ cells, which is superior to that of the *cKit*-*Cre*, *Pgk2*-*Cre*, *Hspa2*-*Cre*, and *Syn*-*Cre* mouse strains reviewed in detail by Lee Smith [19] (Table 1).

### 3.3. Wisp3-Cre

*Wisp3*, activated by the Wnt-1 signaling pathway, is involved in multiple cellular physiological functions and carcinogenesis, including colon and breast cancers. Its mutation is related to progressive pseudorheumatoid arthritis, which is a type of autosomal recessive hereditary disease [86]. However, the mutation or overexpression of *Wisp3* in mice results in no visible phenotypes compared to wild-type animals [87]. To further investigate the physiological actions and spatial expression of *Wisp3* in mice, the *Wisp3-GFPCre* KI mouse line was established, with *GFPCre* inserted into the first exon of *Wisp3*, replacing *Wisp3* expression with GFP and *Cre*. *Cre* recombination activity has been reported by mating with females carrying the *ROSA26^mTmG^* allele. Higher levels of *Cre*-mediated recombination were observed only in the testes, especially during the prophase of meiosis I in spermatocytes. The recombinant offspring accounted for 7% of those born to females with two double heterozygotes with no recombination activity in the ovary [88]. In addition, William et al. concluded that Aurora A Kinase (AURKA) plays a role in spermatid physiology and mouse fecundity using the *Aurka* spermatocyte KO mouse strain, generated by crossing *Aurka^fl/fl^* female mice with one floxed allele with *Wisp3*-*Cre* transgene male mice [89]. In conclusion, although *Wisp3* is not essential for mouse fertility, the male mice carrying the *Wisp3*-*Cre* allele contribute to exploring the role of genes in the meiosis I stage of spermatogenesis.

### 3.4. Spo11-Cre

DNA double-strand breaks (DSBs) are crucial for initiating meiotic recombination and are initiated by SPO11. *Spo11* mutation causes a synaptic defect in pachytene and leads to meiotic arrests in both males and females, resulting in the apoptosis of spermatocytes and oocytes [90]. Manuela et al. determined the physiological function of *JAM-C* in the germ cells of a cKO model in transgenic mice expressing *IRES*-*Cre* driven by *Spo11* during early meiosis [91]. Lyndaker et al. also generated *Spo11*-*IRES*-*Cre* mouse tools (Strain #:032646) to investigate the role of HUS1 in DSB repair during meiotic prophase I (Figure 2) [92]. However, the transgenic *Spo11* locus not only includes the entire *Spo11* promoter sequence but also fuses with the *IRES* sequence upstream of the start site [91,92]. Further studies found that the expression of *Spo11* in transgenic mice was disrupted by negative feedback, leading to a significant reduction in the *Spo11* level and a direct and substantial impact on the meiotic process [93]. The Jordan group found that the main defect in spermatogenesis occurs in spermatocytes rather than in spermatids after *Aurka* mutation in *Spo11*-*IRES*-*Cre* cKO models [92,94]. They did not note that endogenic *Spo11* function is disturbed by *Cre* recombination, which differs from the results obtained by William et al., who concluded that *Aurka* is required not only for spermatocyte maintenance but also for spermatid morphogenesis using *Aurka* spermatocyte KO mice excised by *Wisp3*-*Cre* [89]. In summary, the use of *Spo11*-*Cre* in the study of gene functions in spermatocytes is limited and has rarely been reported.

## 4. *Cre* Recombination Strains for Spermatids

### 4.1. Tspy-Cre

Human *Tspy*, localized on the Y chromosome, encodes proteins expressed in the testis and is conserved in placental mammals, including artiodactyl [95], rodents [96], and perissodactyl [97]. *Tspy* functions in the cell cycle with differentiation, indicating its role in spermatogonial development [98]. As *Tspy* was silenced naturally in experimental mice, human *Tspy*, including a 2.8 kb coding region with a 2.95 kb promoter region, was used to establish a transgenic mouse line to analyze *Tspy* physiological characteristics in the testis [99]. Mice harboring human *Tspy (hTspy)* presented normal phenotypes, and the counts of pachytene spermatocytes and spermatids did not change sharply compared to wild-type controls [99]. Furthermore, the *hTspy*-*Cre* recombinant mouse was constructed in the control of a 2.4 kb *hTspy* promoter, and it was found that the *hTspy* recombination activity was mainly in round and elongated spermatids, as shown by EGFP immunostaining in the progenies born from double *hTspy*-*Cre*/*Z/EG* mice expressing EGFP, especially in the testis [100]. Additionally, EGFP is expressed in the ovary and in the central and peripheral nervous systems as early as E12.5. Thus, the *hTspy*-*Cre* transgenic mouse line can be used as a model for exploring gametogenesis. However, *Cre* activation in multiple tissues may result in an inaccurate determination.

### 4.2. Prm1-Cre

Endogenous protamine is expressed in the haploid stages of spermatogenesis [101], and exogenous genes fused with the *Prm1* proximal promoter are primarily limited to haploid spermatids [102,103] despite lower ectopic expression in the heart and temporal bone [104]. One type of *Prm1*-*Cre* mouse line (Strain #:003328) comprises a fusion of *Cre* with the 652 bp fragment of the mouse *Prm1* promoter. Recombination efficiency was primarily observed in male germ cells with non-significant functionality both in embryonic stem cell lines and somatic tissues from embryos or adult mice [17]. However, the recombination efficiency of *Prm1*-*Cre* is doubtful, with only about 50% reported when flox-flanked *Pofut1* and *Mgat1* were silenced, as noted by Frank et al. [23]. Another *Prm1*-*Cre* mouse line developed by Schmidt et al. demonstrated the catalytic activity of *Prm1*-*Cre* recombinase in post-meiotic spermatids. However, all paternal *Cre*-bearing mice and *Cre*-modified male offspring modified by *Cre* from female founders were infertile. Further analysis revealed that 100% of abortive pregnancies were caused by spermatid chromosome rearrangements catalyzed by *Cre* recombinase in embryos acquired from wild-type females crossed with males carrying the *Cre* gene [105]. 

### 4.3. Acrv1-iCre

*Cre* recombinase specific to spermatids was established to be limited to haploid cells after meiotic division. Offsprings from mating floxed transgenic and heterozygote *Cre* recombination strains are the result of an inevitable part of haploid sperm being recombination-deficient without exhibiting presumptive phenotypes. Theoretically, approximately half of non-recombining sperm hold some promise for normal reproduction. Thus, increasing recombination efficiency is essential for targeting genes of interest in spermatids. Recently, an *Acrv1*-*iCre* mouse line was genetically engineered using CRISPR/Cas9 to selectively excise genetic segments in spermatids [106]. The expression of *iCre*, initiated by the promoters of *Acrv1*, which is specifically localized in stage 5−8 spermatids, efficiently deleted the floxed allele by more than 97%, as quantified by assessing the percentage of progeny with intact floxed alleles or deletion upon the mating type of *Rosa26^Acrv1-iCre/+^* with *Dot1*f*^fl/fl^* [106]. The generation and characterization of the *Acrv1*-*iCre* recombination line by Julie et al. [106] contradicted the hypothesis that a small number of alleles in haploid spermatids would not be knocked out because of the common crossing approach. We assumed that *Cre* transcripts already functioned either prior to or during the second meiotic division process. *Cre*-negative spermatids can activate recombination activity through intercellular communication, such as exocytosis [107], which coincides with the concept that *Cre* can pass between haploid spermatozoa via cytoplasmic bridges [19]. Moreover, *Cre* transgenic mouse lines offer an advantageous technique for establishing global KO models to investigate large quantities of gene sequences in somatic tissues if normal reproductive behavior is observed after the genes in either early germ cells or haploid spermatozoa are excised.

### 4.4. Elf5-Cre

ELF5, an ETS transcription factor, is localized in the trophoblast lineages of the embryo as well as in the prostate, kidney, lungs, testes, and mammary gland during postnatal development [108,109,110]. Shuangbo et al. established an *Elf5*-*Cre* transgenic mouse line by co-injecting the constructed *2A*-*Cre* coding sequence with Cas9/sgRNA into the pronuclei, and the *Cre* sequence was integrated into the exon of *Elf5* close to the stop codon to investigate the contribution of genes in placental development (Figure 2) [108]. Typically, *Cre* is activated in all trophoblast-derived lineages. Furthermore, a robust signal was detected in spermatids and sperm, suggesting a novel transgenic mouse strain for functional gene studies confined to the late stage of spermatogenesis [108]. However, due to the catalytic activity of *Elf5*-*Cre*, attention should be paid to its specificity and efficiency when studying genes of interest in haploid sperm. 

**Table 1:** Characteristics of *Cre* models generated for male germline research.

Marker Strains	Germline Specific	Expression Outside of the Reproductive System	Initial Expression Phase	Transgenic (Tg)/Knock-In (KI)
*Oct4*-*MerCreMer* [78,79] (Strain #016829)	PGCs and undifferentiated spermatogonia	Pancreas, skin, intestine, kidney, etc. [80]	E7.5–8 [77]	KI
*Tnap*-*Cre* [66]	PGCs (around 50%)	Placenta, intestine and neural tube, labyrinthine region	E9.5–10.5	KI
*Nanos3*-*Cre* [67]	PGCs (11–25%)	NR	E7.75	KI
*Nanos2*-*MerCreMer* [111,112]	undifferentiated spermatogonia	NR	E13.5 [113]	Tg
*Blimp1*-*Cre* [68,114] Strain #008827	PGCs (55–78%)	B and T lymphocytes, retina, limbs, pharynx, and heart [70]	E6.25	Tg
*Tex101*-*iCre* [115,116] Strain #019893	Pro-spermatogonia and subsequent germ cells	NR	1 DPP	Tg
*Gfra1*-*CreER^T2^* [117]	Undifferentiated spermatogonia	Kidney [118]	E9.5	KI
*UBC*-*CreER^T2^* [71,72,74,75,119] Strain #:007001	Spermatogonia, testis, and somatic cells	Thymus, spleen, heart, muscle, brain, kidney, bone marrow [119]	NR	Tg
*Rosa26-CreER^T2^* [73]	Spermatogonia	Other tissues in embryo and adult [120]	NR	KI
*Aqp2*-*Cre* [121] Strain #:006881	Spermatids	Kidney	NR	Tg
*Hspa2*-*Cre* [122,123] Strain #:008870	Spermatocyte and spermatids	Brain and embryo	Leptotene	Tg
*Pgk2*-*Cre* [124,125]	Spermatocyte and spermatids	Tissues in embryo [125]	NR	Tg
*Wnt7a*-*Cre* [126,127] Strain #036637-JAX	Spermatocyte	Uterine epithelium [127]	Mid-pachynema (12 DPP)	Tg
*cKit*-*Cre* [128]	Spermatocytes and spermatids	Mosaicism (20–100%)	NR	Tg
*CaMKIIα*-*Cre* [129,130] Strain #:005359	Testis germ cells	Brain	NR	Tg
*Syn1*-*Cre* [131,132] Strain #:003966	Spermatocytes	Neurons [132]	E12.5	Tg

NR: not reported.

## 5. *Cre* Transgenic Mice for Sertoli and Leydig Cells

*Cre* lines specific for Sertoli cells, including *Abp*-*Cre*, *Amh*-*Cre* (Strain #:007915), *Dhh*-*Cre* (Strain #:012929), and *Dmrt1*-*Cre*, have been used extensively and were described by Smith [19]. Here, novel *Cre* lines specific to Sertoli cells and their practical applications are described. *Sry* was exclusively detected in the supporting cells of genital ridges during E10.5–E12.5 and mediated the fate of supporting cells towards Sertoli cells [133]. *Sry*-*Cre* compromising the 9.9 kb *Sry* sequence under the controls of 5′ and 3′, untranslated regions of endogenous *Sry*, was constructed to explore the fate of *Sry*-positive cells [134] as well as the functional region of sex determination-related genes [135,136]. *Sox9* is expressed in multiple tissues and cells, including the CNS, intestine, and Sertoli cells, and regulates cell growth and differentiation during mouse embryogenesis. The *Sox9*-*Cre* transgenic line was generated by fusing *Cre* recombinase with an internal ribosome entry sequence and knocking it into the *Sox9* locus at the 3′ untranslated region [137]. The systematic analysis of *Sox9*-*Cre*/*R26R* mice revealed that *Sox9*-positive cells, as progenitors, were conducive to a variety of cell types, including chondrocytes, Leydig cells in the testis, intestinal epithelial cells, and all cells in the pancreas and spinal cord of the mouse embryo from E8 to E17 [137]. Therefore, the *Sox9*-*Cre* mouse line can be used for sex differentiation investigations because of its catalytic activity in early embryos. Yayoi et al. established a *Sox9*-*Cre*/*Nr5a1^fl/fl^* mouse strain and concluded that *Nr5a1* plays a crucial role in mouse gonadal sex determination [138]. *Sox9*-*CreER* mice (Strain #:035092) were generated after *IRES*-*CreER^T2^*-*SV40pA* cassettes integrating into endogenous *Sox9* at 3′UTR by the Cas9/RNA targeting method and have been applied in embryonic lineage tracing experiments after mating with *Rosa*-*stop*-*mTmG* mice [139]. However, *Sox9*-*Cre* was restricted in its application to males due to its recombinant activity in a broad range of tissues.

*Cyp17-iCre* (Strain #:028547) and *Cyp11a1*-*iCre* in Leydig cells were reviewed in 2011 with ectopic expression in the brain and adrenal glands [19]. Additionally, a second *Cyp11a1*-*iCre* transgenic line was generated by BAC construction comprising a 2.8 kb *Cyp11a1* and *iCre* sequence, and the *iCre* catalytic activity was controlled by the mouse promoter [140]. Laura et al. established another novel mouse line, *Cyp11a1*-GC, with dual characteristics: it not only silenced the endogenous *Cyp11a1* function but also simultaneously knocked-in *Cre* recombinase, which excises the genes of interest in steroidogenic cells without changes in the circulating testosterone concentration [141]. Annalucia et al. characterized the ability of viral vectors, including adenovirus, lentivirus, and adeno-associated virus (AAV), to deliver exogenous genes targeting Leydig cells in adult mouse testes and determined that AAV serotype 9 (AAV9) + neuraminidase transported the transgenes efficiently [142]. Moreover, AAV9-driven *Cre* was generated by the Diane group to delete endogenous glucocorticoid receptors in adult Leydig cells; the silencing efficiency of AAV9-*Cre* to AR was 48% 7 days after injection, which was higher than that of the *Cyp17a1*-*iCre* transgenic approach (28%) [143]. The AAV9 virus was found to infect germ cells of the testes from 3-week mice [144], possibly because these testes are not fully developed in immature mice, and the blood–testis barrier is not fully established. Thus, the AAV9-*Cre* silencing method is more suitable for exploring the genetics underlying the functions of adult Leydig cells, whereas the *Cyp17a1*-*iCre* model is more suitable for functional studies of developing Leydig cells.

The type II *Amh* receptor, *Amhr2*, is localized in the mesenchyme of the Müllerian duct, Leydig cells, Sertoli cells, and granulosa cells and initiates the degeneration of Müllerian ducts after binding with the anti-Müllerian hormone [145]. The *Amhr2*-*Cre* line (B6;129S7-*Amhr2^tm3(cre)Bhr^*/Mmnc from the Mutant Mouse Regional Resource Centers) was generated by knocking in the targeting vector to the endogenous *Amhr2* loci (Figure 2) [146]. The catalytic activity of *Amhr2*-*Cre* was observed in both the Leydig and Sertoli cells of the testis, theca cells, and granulosa cells in the ovary [147,148]. The *Amhr2*-*Cre* mouse model has also been used as a genic toolkit for the study of testicular granulosa cell tumors [149]. Notably, *Amhr2*-*Cre* mice mating with (translocator protein) *Tspo*-floxed mice generated global *Tspo* KO mice instead of the *Tspo* cKO line [24], probably because of the genetic linkage of *Amhr2* with *Tspo*, which are expressed together during the early embryo stage. Thus, emphasis should be placed on whether global KO occurs when using the *Amhr2*-*Cre* mutation approach. Furthermore, the Chauvin group, collaborating with the Jackson Laboratory, developed *Amhr2*-*CreER^T2^* mice (Strain #:037056) by the CRISPR/Cas9-mediated approach, which has been explored in determining the fate of cancer-associated mesothelial cells in ovarian cancer upon *Amhr2* induction by crossing with the *ROSA26^mTmG^* mouse line [150]. The recombination efficiency of this novel mouse line requires further evaluation.

## 6. *Cre* Transgenic Models for Other Cells in the Testes

In addition to spermatogenic, Leydig, and Sertoli cells, various other cell types have been well established in the testes, including T cells, endothelial cells, peritubular myoid cells, mesenchymal (stromal) cells, macrophages, tenocytes, two pericyte subpopulations (with either smooth muscle or ECM-secreting properties), and a Leydig cell precursor population [151,152,153]. However, the functional characteristics of these cells contributing to spermatogenesis are yet to be elucidated, and their protein expression patterns have not been identified. Several generalized *Cre* lines were used to investigate the gene function in these cells. *smMHC*-*Cre* (also called *Myh11*-*Cre*) (Strain #007742) for vascular smooth muscle cells and peritubular myoid cells (PTM) and *SM22*-*Cre* lines (Strain #:004746) for PTM were described by Smith [19]. *Myh11*-*Cre* was used to uncover the multiple functions of genes in testicular PTM. The synthesis of the testosterone-dependent glial cell line-derived neurotrophic factor (*GDNF*) in PTM cells was found to maintain the microenvironment of the spermatogonial stem cell (SSC) niche, together with Sertoli cells [154]. The initial recombination activity of the *Myh11*-*Cre* mouse line was detected at E12.5, and *Cre* efficiency was observed in all smooth muscles of adult mice, including the heart, bladder, lung, and testes [155]. The essential roles of *GDNF* [156] and *Gops5* [157] in PTM cells on mice reproductive function and postnatal development in PTM cells have been demonstrated in *Myh11*-*Cre* cKO models. Furthermore, tamoxifen-inducible *Myh11*-*CreER^T2^* or *Myh11*-*DreER^T2^* mouse strains virtually eliminated specific targets in PTM cells [158]. However, *Myh11*-*Cre* expression in the testes was not restricted to PTM cells and was also found in blood vessels; thus, interference may occur in the prospective phenotype in cKO mice [159]. Moreover, *Cxc3* chemokine receptor 1 (*Cx3cr1*) was found in monocytes and resident macrophages in all mouse tissues [160]. *Cx3cr1*-*Cre* mice were generated and used for the conditional expression of the diphtheria toxin receptor in testis macrophages for further excision, despite no direct evidence of a cKO event, with an efficiency of approximately 95%, indicating the effectiveness of *Cx3cr1*-*Cre* in the macrophage population [160,161]. However, *Cx3cr1* is extensively expressed in macrophages and monocytes; therefore, potential disturbances cannot be ignored.

Evidence suggests that a *Cre* line is generated, particularly targeting endothelial cells of the testis. *Tie2*-*Cre* (Strain #:008863) is localized in endothelial cells in the testis [19,162]. O’Hara et al. performed further research by applying *Tie2-Cre* to inactivate androgen receptors in testicular vascular endothelial cells [163]. Additionally, *Cre* lines that have undergone careful validation in other tissues are expected to remove the targets of interest in endothelial cells of the testis, such as *Cdh5*-*Cre* [164] and *Pdgfb*-*iCreER^T2^* [165]. Concerns regarding *Cre*-engineered mouse strains, especially macrophages, have been widely reported, with *LysM*-*Cre* and *hCD68*-*CreER^T2^* being the common methodologies [166]. *LysM*-*Cre* is prominently expressed in major myeloid lineage cell types, such as monocytes and neutrophils (Figure 2), whereas *hCD68*-*CreER^T2^* primarily targets tissue-resident macrophages and is inducible by tamoxifen [167]. *LysM-Cre* was utilized to generate myeloid-specific ubiquitin-specific protease 2 (USP2) cKO mice, revealing the necessity of macrophage *USP2* for sperm physiological functions, including motility, capacitation, and hyperactivation [168]. Despite reports of the recombination efficiency of *Cre*, evidence supporting its ability to eliminate specific genes in testicular macrophages remains lacking. Other cell types identified through single-cell sequencing in the testes, such as T cells, mesenchymal (stromal) cells, and tenocytes [151,152,153], have been poorly studied in terms of their regulation of germ cell development, and a detailed description of the cKO strategy is not provided.

## 7. *Cre* Models for Epididymis

Sperm exhibit progressive motility and fertilization properties during transit in the epididymis, a highly convoluted duct comprising four main anatomical parts as follows: the initial segment (IS), caput, corpus, and cauda, each with distinct regional functions and characteristics [169]. The microenvironment of the epididymal lumen undergoes various changes during sperm transportation, with a large number of ions, proteins, and miRNAs absorbed or released into the lumen fluid [169]. Approximately 40% of male idiopathic infertility cases are associated with defects in sperm maturation, underscoring the importance of the epididymal function in sperm maturation [170]. Although assisted reproductive techniques (ART) contribute to improving sperm maturation status, reproductive risks related to artificial interventions are increasingly emphasized [170]. It is necessary to elucidate the mechanism of sperm maturation in the epididymis and ameliorate male reproductive performance with minimal external interventions. To date, multiple *Cre*- engineered mouse models have been employed in many investigations of targets in the epididymal segments.

### 7.1. Defb41-iCre

DEFB41, containing 62 amino acids, is a specific beta-defensin, primarily localized in the epithelial cells of the initial segment and caput, with weak signals in the prostate, corpus, and pancreas [171]. The initial expression was detected at 7−14 DPP, peaked at 25 DPP, and remained stable after 40 DPP [172]. The *Defb41*-*iCre* KI mouse line was generated using the red/ET recombination approach, and the first exon of *Defb41* was inserted with *Cre* recombinase constructed in a BAC [172]. Björkgren et al. showed that *Defb41* ablation in the epididymis altered sperm progressive motility and the ability to bind to the oocyte, whereas sperm morphology and count were unaffected in the homozygous *Defb41^iCre/iCre^* mouse strain [172]. Consequently, heterozygous *Defb41^iCre/+^* mice were used as a cKO tool line to detect *Dicer1* physiological properties in the initial segment and caput by the progeny from Dicer1*^fl/fl^*; *Defb41^iCre/+^* mice [173,174].

### 7.2. Rnase10-Cre

The initial expression of *Rnase10*, coinciding with IS differentiation, was detected at approximately 17 DPP [175]. The disruption of proximal proteins encoded by *Rnase10* in the mouse epididymis is associated with a penetrating defect in the zona pellucida, rendering sperm unable to pass through the female uterotubal junction district [176]. The *Rnase10*-*iCre* line was established by introducing the *iCre*-*NeoR* cassette into the *Rnase10* translation initiation locus in the first eight nucleotides of exon 2 [177]. The androgen receptor (AR) was inactivated by the *Rnase10*-*iCre* mice mating with *AR^fl/fl^* homozygous females, validating the critical role of AR in the function and development of IS [177].

### 7.3. Crisp4-Cre

CRISP4 is a cysteine-rich secretory protein (CRISP) that is highly expressed in murine principal cells in the epididymal epithelium, with its transcripts most abundantly present in the caput and corpus and few signals in the thymus and spleen [178,179]. CRISP4 has also been detected in epididymosomes and seminal plasma [180] and is strongly associated with sperm maturation during the epididymal transport process [181]. CRISP4 inactivation causes the failure of protein tyrosine phosphorylation and the acrosome reaction induced by progesterone in the capacitation process [182] and is incapable of fertilization with zona pellucida-intact eggs, probably due to the calcium channel TRPM8 defect and failure to regulate the acrosome reaction [181]. *Crisp4*-*iCre* KI mice were generated by inserting the *iCre*-neomycin phosphotransferase cassette into the third *Crisp4* exon locus before the initiation codon, resulting in a transcription frameshift. The homozygous *iCre*-recombinase line acts as a *Crisp4*-deficient model, and the heterozygous mouse model can be used to excise targets of interest in the epididymis [180]. *Crisp4*-*iCre* is expressed in the epididymis at day 20 of postnatal development, and its level increases with age [180]. The specificity of the *Crisp4*-*iCre* recombination event performed by *Crisp4*^+/−^/*Z/RED^+^* and *Crisp4^+/^*^−^/*Runx1^fl/+^* transgenic mice was expressed in the whole epididymis tissue and in proximal epididymis without signals in other tissues detected, respectively [180], indicating that *Crisp4*-*iCre* is capable of ablating targets in vivo.

### 7.4. Lipocalin-Cre

mE-RABP (*Lcn5*), mEP17 (*Lcn8*), and lipocalin 9 (*Lcn9*), defined in the lipocalin family by phylogenetic analysis, are murine secretory proteins localized in the epididymis [183]. *Lcn5* is synthesized in principal cells residing in the middle/distal caput regions and is initially secreted from 30 DPP, with a gradual increase to 60 DPP [184]. *Lcn8* and *Lcn9* are positioned in the IS with similar expression patterns and are expressed at 21 DPP during postnatal development, depending on testicular factor regulation [183,185]. Spermatogenesis and fertility were normal conditions after *Lcn8* or *Lcn9* inactivation in the epididymis, while *Lcn8* ablation caused an increased teratospermia rate, sperm motility, and acrosome reaction frequency deficiency, indicating its indispensable role in sperm maturation [186]. Lipocalin family genes are highly conserved and show homology among subtypes, with the probability of functional overlap in a physiological manner. The transgenic lines in *Lcn5*, *Lcn6*, *Lcn8*, and *Lcn10* were knocked out simultaneously, or *Lcn5*, *Lcn6*, *Lcn8*, *Lcn10*, and *Lcn9*, when silenced synchronously, showed subfertility and infertility in most cases [187].

The *Lcn5*-*Cre* transgenic mice were established by Xie et al. using the *Cre*/*LoxP* system, where *Cre* activity was regulated by the *Lcn5* promoter (1.8 kb). Initially, *Cre* catalytic activity was observed at 30 DPP, showing high specificity in middle or distal caput principal cells when crossed with the reporter strain mT/mG or with *the Aip1^fl/+^* mouse line. The recombination efficiency was statistically significant at 28.9% [188]. A tamoxifen-sensitive *Lcn5*-*CreER^T2^* transgenic line was generated by the same group. *Cre* activity was also highly restricted in the caput epididymis by tamoxifen induction in a time- and dose-dependent manner, providing an approach for the further analysis of the spatiotemporal functions of target genes in the caput epididymis [189]. *Lcn8*-*Cre* or *Lcn9*-*Cre* mice were generated by the insertion of the *NLS*-*Cre*-*polyA* or *2A*-*NLS*-*Cre* cassette into the *Lcn8* or *Lcn9* promoters, respectively, using CRISPR/Cas9 technology; *Cre* expression led to the loss of *Lcn8* and *Lcn9*. *Lcn8*-*Cre* and *Lcn9*-*Cre* activity is specially confined to the principal cells of the IS, without reproductive disorders, in adult male mice [190,191]. Novel *Lcn8*-*Cre* and *Lcn9*-*Cre* models can be used to conduct functional gene studies that are specific to IS segments (Figure 2). However, transgenic mouse models with *Cre* bioactivity in the epididymal cauda are still lacking; thus, generating more recombinant mouse lines, particularly those with silencing targets in the epididymis, is necessary.

## 8. The *Cre* Models Generated for the Prostate, Seminal Vesicle, and Seminiferous Duct

The prostate is an adnexal gland of the male genitourinary system, primarily comprising the stroma and epithelium. Prostatic fluid is secreted by the prostate epithelial compartment and occupies approximately 1/5 to 1/3 of the volume of ejaculated semen. It contributes to sperm motility, the clotting cycle, and semen liquefaction [192]. *Cre* expression under the regulation of the promoter derived from the prostate-specific probasin (*ARR2PB*) of rats and *ARR2PB*-*Cre* (Strain #:026662) catalytic activity was detected predominantly in all lobes of the mouse prostate. The highest signals were observed in the lateral lobes driving androgen-dependent transcription events, while *Cre* activity was least visible in the anterior and dorsal lobes. The further functional characterization of *ARR2PB*-*Cre* was carried out by selectively removing *RXRa* from the mouse prostate and avoiding the embryonic lethality caused by *RXRa* global KO [193]. In addition, Jin et al. generated an *ARR2PBi*-*Cre* transgenic mouse line (Strain #023325), showing uniform expression across all lobes of the prostate as well as in the ductus deferens and seminal vesicles. This model exhibited higher efficiency in interfering with gene activity in target tissues [194]. *ARR2PBi*-*Cre* mice are a valuable tool for genetic-based investigations of the prostate, seminal vesicles, and ductus deferens (Figure 2).

## 9. Conclusions and Perspective

An orchestrated series of events, mainly involving PGC development, SSC maintenance, spermatogenesis, sperm maturation, and ejaculation, ensures male fertility under normal conditions depending on certain genetic expressions during different physiological phases. Conventional KOs often lead to embryonic or perinatal lethality, particularly in homozygotes, and may result in unpredictable impacts on other organs with abnormal physiology. The increasing use of *Cre* recombination lines highlights the validity of the *Cre*/*LoxP* system as a valid molecular genetics approach for reproductive research. In this study, we summarized the *Cre* mouse lines used to investigate gene behavior during various processes related to germ cell biology and sperm physiological function. We also addressed existing issues such as unstable efficiency, global KO, the ectopic expression of *Cre* activity, and strong maternal effects. The unpredictable expression pattern of *Cre* in the testes may be attributed to chromatin compaction and rearrangement during meiosis, as well as the randomness of insertion sites and cassette defects in traditional gene recombination methods. As early as 2013, the CRISPR/Cas9 system was used to mutate the mouse genome, resulting in a genetically disrupted mouse line that was achieved faster and at lower costs [13]. CRISPR/Cas9 can act on multiple loci simultaneously and contribute to investigating the combined effects of genes on reproduction or establish connections among targeted genes in the fertility process [195,196]. Moreover, random insertions and uncontrolled gene copies caused by transgenic steps in conventional recombination are avoided in the CRISPR/Cas9 system [197]. In this review, we summarized several gene-disrupted mouse lines edited by the CRISPR/Cas9 approach, including *Ddx4*^i*Cre*^ [15], *Ddx4*^em1(*CreERT2*)Utr^ [20], *Stra8*^P2A-*Cre*^ [43], *Acrv1*-i*Cre* [106], *Lcn8*-*Cre* and *Lcn9*-*Cre* [190,191], which showed higher specificity, efficiency, and the lower occurrence of global KO events. Other recommendations are as follows: 1. Combining multiple inducible *Cre* lines can help inactivate target genes localized in distinct regions of the epididymis, as some genes are distributed throughout the entire epididymis without regional differences, and a clear phenotype may not be visible under gene disruption in a single segment. 2. The strict control of *Cre*-*LoxP* can be strengthened by combining it with the *Dre*-*Rox* system, making lineage tracing more precise and minimizing the non-determinate effects of *Cre* catalytic activity on certain cells [198]. Thus, a more credible and accurate in vivo study of the cell lineage from the male reproductive system is warranted by this novel technology, as it greatly reduces the nonspecific recombination of the conventional *Cre*-*LoxP* system. 3. There is a demand to generate new homozygous Cre lines for gene recombination in spermatids with higher efficiency and stable outcomes, and it is worth considering the expansion of *Cre* models for the corpus and cauda epididymis. 

Overall, as a large number of genes play a vital role during the male reproductive process, more mouse tool models can be established and be made available for detailed descriptions of genes to reveal the male reproductive mystery at the genetic level.
